# Functional and aesthetic outcome of different methods of reconstruction of full thickness lip defects

**DOI:** 10.3205/iprs000163

**Published:** 2022-03-08

**Authors:** Asif Iqbal Shaikh, Arshad Hafeez Khan, Sushrut Tated, Naveen Khubchandani

**Affiliations:** 1 Department of Plastic Surgery, Jawaharlal Nehru Medical College and Hospital, Aligarh (UP), India

**Keywords:** lip reconstruction outcome, aesthetic, functional, microstomia, lip flaps, Karapandzic flap, Estlander flap, lip advancement, nasolabial flap, full thickness lip defect

## Abstract

**Background:** The lip has functional and aesthetic importance. Lip defects occur due to the variety of etiology and the choice of their reconstruction has profound effect on functions and cosmesis. There are multiple options available for reconstruction according to defect size, but superiority of one method over another is still debated and hence the methods and their outcome were analyzed prospectively.

**Material and method:** Twenty-one patients with all sizes and locations of defects in upper and lower lip with acquired etiology were included in the evaluation. Reconstruction was performed according to defect size, availability of local/regional and distant donor tissue, defect location, patients’ comorbid conditions and patients’ preference. Patients were assessed at 1 month and 6 months postoperatively. Observers’ and patients’ input were also taken into account for outcome.

**Results:** Out of 21 patients, 5 free radial artery forearm flap reconstructions, 4 nasolabial flap reconstructions, 5 primary closures of defect, 4 Estlander flap reconstructions, 2 lip advancements, and one Karapandzic flap reconstruction were done. Free flap and nasolabial flap had hypoesthesia and incompetence if commissure is reconstructed and problem of bulk, restricted mobility and vermilion mismatch. Local and lip flaps were associated with decreased stoma size and some form of local scarring and asymmetry. However, all patients were satisfied with the functional and aesthetic outcome.

**Conclusion:** Local flaps are better in terms of functional and aesthetic outcome but with some degree of microstomia which was well tolerated by most patients. Regional and distant flaps provide reconstruction where no other option is available and provide good functional support and acceptable cosmesis.

## Zusammenfassung

**Hintergrund:** Die Lippe hat funktionelle und ästhetische Bedeutung. Lippendefekte treten aufgrund unterschiedlicher Ätiologie auf und die Wahl ihrer Rekonstruktion hat tiefgreifende Auswirkungen auf Funktionen und Kosmese. Es gibt mehrere Optionen für die Rekonstruktion je nach Defektgröße, aber die Überlegenheit einer Methode gegenüber einer anderen wird immer noch diskutiert, daher wurden die Methoden und ihr Ergebnis prospektiv analysiert.

**Material und Methode:** 21 Patienten mit allen Größen und Orten von Defekten der Ober- und Unterlippe mit erworbener Ätiologie wurden in die Untersuchung einbezogen. Die Rekonstruktion wurde nach Defektgröße, Verfügbarkeit von lokalem/regionalem und entferntem Spendergewebe, Defektort, Komorbiditäten der Patienten und Präferenz der Patienten durchgeführt. Die Patienten wurden 1 Monat und 6 Monate nach der Operation untersucht. Eingaben der Beobachter und der Patienten wurden ebenfalls für das Ergebnis berücksichtigt.

**Ergebnisse:** Bei den 21 Patienten wurden 5 Rekonstruktionen mit freiem Radialis-Unterarmlappen, 4 Rekonstruktionen mit Nasolabiallappen, 5 primäre Defektverschlüsse, 4 Rekonstruktionen mit Estlander-Lappen, 2 Lippenvorschübe und 1 Rekonstruktion mit Karapandzic-Lappen durchgeführt. Bei freiem Lappen und Nasolabiallappen traten Hypästhesie und Funktionseinschränkung auf, wenn die Kommissur rekonstruiert wurde, und das Problem der Größe, der eingeschränkten Mobilität und der Rot-Fehlanpassung. Lokale Lappen und Lippenlappen waren mit einer verringerten Stomagröße und einer Form von lokaler Narbenbildung und Asymmetrie verbunden. Alle Patienten waren jedoch mit dem funktionellen und ästhetischen Ergebnis zufrieden.

**Schlussfolgerung:** Lokale Lappen sind hinsichtlich des funktionellen und ästhetischen Ergebnisses besser, weisen jedoch einen gewissen Grad an Mikrostomie auf, der von den meisten Patienten gut akzeptiert wurde. Regionale und entfernte Klappen erlauben eine Rekonstruktion, wenn keine andere Option verfügbar ist, und bieten eine gute funktionelle Unterstützung und eine akzeptable Kosmese.

## Introduction

The lip has complex functions including expression of feelings and smiling, oral sphincter function, articulation, and serving as the symbol of aesthetic beauty. Complete competence, maximum oral opening, mobility, sensation, and maximum aesthetic results should be the goals of the reconstruction undertaken [1].

There is a general agreement that defects of one third or less of the lips are repaired by primary closure with variations of incisions with excellent functional and cosmetic outcome [2], [3]. For larger lip defects, lip reconstruction gets more complicated and a search for the ideal method of reconstruction has brought forth a large number of different methods and techniques of which each has its own advantages and disadvantages [4], [5], [6], [7], [8], [9]. More than 200 different methods of lip reconstruction techniques, varying from primary closure to free-flap transfer, have been described since the mid-19^th^ century. The dimensions and the localization of the defect play important roles in the selection of the type of reconstruction.

To assess the functional and aesthetic outcome of different methods of reconstruction of full thickness lip defects we have undertaken this analysis.

## Material and method

This prospective analysis was conducted in the plastic surgery department of a tertiary care center from November 2017 to December 2019 after getting clearance from the institutional ethical committee.

Consenting patients of all age groups and both sexes with full thickness lip defect of any size with acquired etiology were included in this. The reconstruction of the lip was done according to the standard operative technique.

Functional assessment was done by assessing oral competence, lip mobility, and sensations. Oral competence was assessed as incompetence, sialorrhea at rest, sialorrhea formed with fluid intake and complete competence. Labial mobility was assessed as symmetric/asymmetric by observing pouting, smiling and mouth opening movements. Sensory assessment included the assessment of all sensations including two-point discrimination. 

Aesthetic assessment was done by examining lip appearance at rest (symmetrical/asymmetrical), size of the lip in a horizontal and vertical direction [status of oral stoma (severe microstomia, moderate microstomia, and normal stoma)], commissure (acute/obtuse), scar aesthetics in nasolabial/mentolabial crease and size of new vermilion to available old residual vermilion. 

Microstomia was assessed subjectively and objectively as shown in Table 1 (Tab. 1).

Two plastic surgeons evaluated the clinical photographs and gave their comment on the aesthetic outcome. The comments were included in final outcome, patients’ comments were also taken into consideration.

## Results

**Figure 1:**
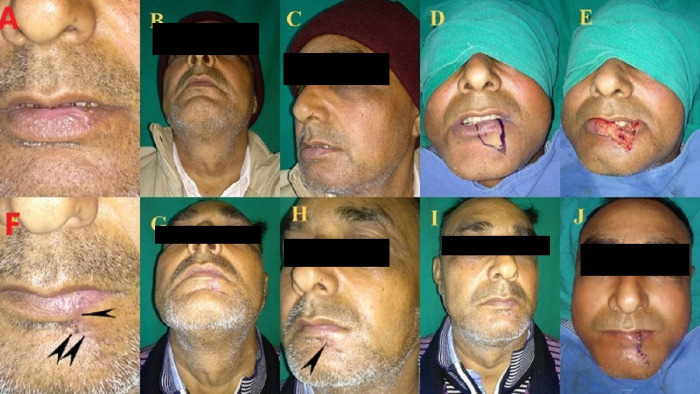
40-year-old male with squamous cell carcinoma lower lip treated with WLE and primary closure. A, B, C, D showing preoporative lip & lesion and marking, E, J showing intraoperative defect and after closure and F, G, H, I showing follow-up. Black arrowheads in F, H showing minimal post-operative scar.

**Figure 2:**
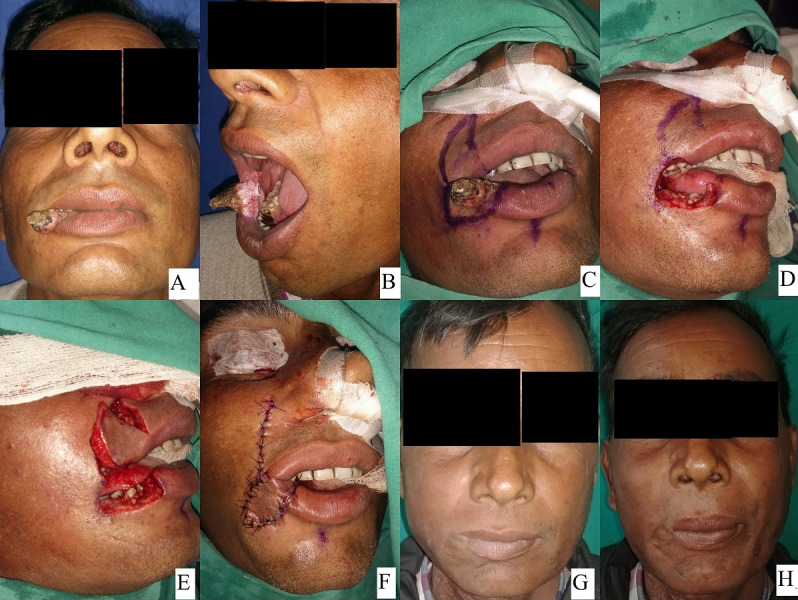
36-year-old male with squamous cell carcinoma left oral commissure treated with WLE with Estlander flap reconstruction. A, B, C showing lesion and marking, D, E, F showing intraoperative defect, flap and in setting. G, H showing follow-up photograph with blunting of right commissure.

**Figure 3:**
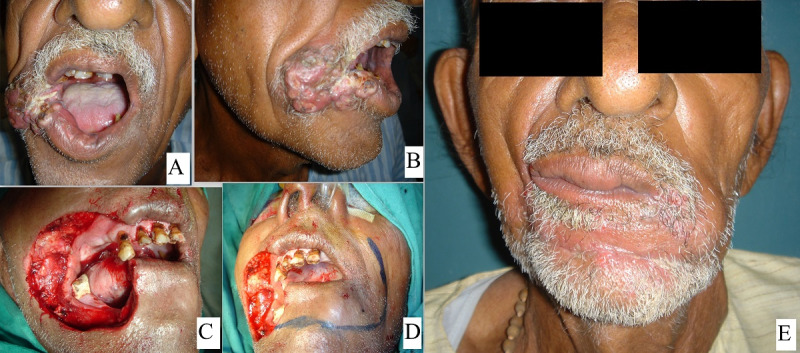
65-year-old male with large commissural and ipsilateral cheek defect treated with unilateral Karapandzic flap. A, B showing lesion, C, D showing defect and flap marking and E showing final follow-up outcome.

**Figure 4:**
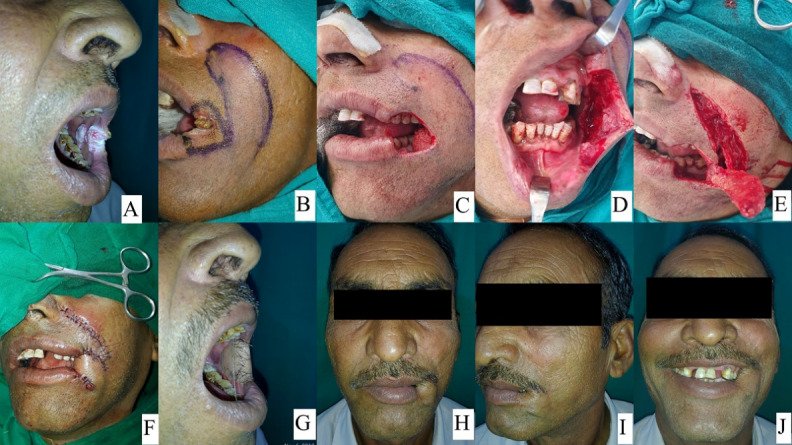
45-year-old male with squamous cell carcinoma left commissure with synchronous lesion in buccal mucosa treated with WLE with ipsilateral nasolabial flap. A, B showing lesion and intraoperative marking, C, D, E, F showing intraoperative defect, flap raised and closed wound, G, H, I, J showing follow-up photograph depicting well settled flap with bulkiness and visible incomplete closure at left commissure and vermillion mismatch.

**Figure 5:**
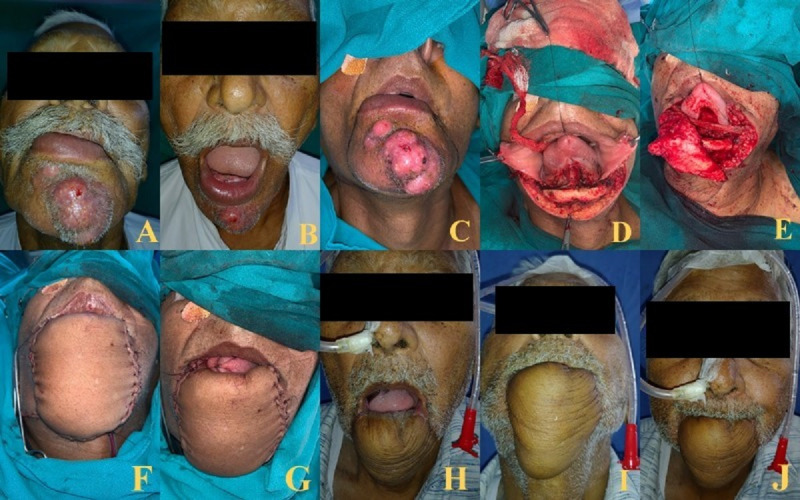
80-year-old male with squamous cell carcinoma lower lip mucosa and infiltrating skin treated with WLE and FRAFF with PL sling. A, B showing lesion, C, D, E, F, G showing marking, defect and flap, H, I, J showing adequate oral opening, flap and competent stoma. Flap bulkiness can be appreciated.

21 patients were operated: 17 males and 4 females with a mean age of 43.29 years and 47.5 years, respectively. Out of the 21 patients 17 patients i.e. 80% of the patients had malignancy, 3 patients i.e. 14.28% had trauma, and one patient i.e. 4.7% had chronic granulomatous disease (TB). Out of the 21 patients, 5 patients had a defect size up to 1/3, 13 patients had a defect size of 1/3 to 2/3 and 3 patients had a defect size >2/3. Out of the 21 patients, 5 patients had primary closure (Figure 1 (Fig. 1)), 4 patients Estlander flap (Figure 2 (Fig. 2)), 3 patients lip advancement and Karapandzic flap (Figure 3 (Fig. 3)), 4 patients nasolabial flap (Figure 4 (Fig. 4)) and 5 patients free radial artery forearm flap (Figure 5 (Fig. 5)).

Table 2 (Tab. 2), Table 3 (Tab. 3) and Table 4 (Tab. 4) show the distribution of the procedures according to extent of lip defect, defect location, and defect size. Nasolabial flap and FRAFF (free radial artery forearm flap) were chosen for two cases in the category of defect size up to 1/3 as there was a buccal mucosal defect along with the lip defect.

Table 5 (Tab. 5) shows the functional and aesthetic outcome of the different procedures.

All local flap patients had a sensory recovery up to 6-month follow-up. One FRAFF patient had sensory recovery with two-point discrimination at 10 mm at 20 months of follow-up. The rest of the patients with no sensory recovery were lost to follow-up after or within 6 months. Incompetence in FRAFF and nasolabial flap may be attributed to commissural defect reconstruction. 

One patient with FRAFF used for reconstruction of commissure with buccal mucosa had a remaining partial lip necrosis which was managed by debridement and primary closure and one patient with bilateral nasolabial flap had a postoperative surgical site infection and partial flap loss which was managed conservatively. For the rest of the patients there were no significant complications.

All patients except one with lip advancement and split-thickness skin graft (STSG) were satisfied with the reconstruction procedure and its outcome.

## Discussion

During lip reconstruction, several outcomes are targeted for. The functionality should be considered first. Without oral competence an esthetically nice reconstruction is bothersome. Oral competence depends on orbicularis oris, with most of its fibers aligned horizontally. Preserving the nerves is also essential because lips with sensitivity are considered a better result than those where sensitivity is not preserved. The best results are obtained by reforming the sphincter and preserving sensitivity [10].

Primary closure of lip defect is associated with microstomia which may be either objective (small defect) or subjective (large defect) but has the advantage of a more anatomical closure with no donor site morbidity, better sensation preservation, competence, and no extra-scarring other than surgical incisions. Color and tissue match is good with the advantage of symmetry preserved in central defect. However, lateral and commissural lesion have problem of asymmetry. According to Soliman et al. [11] full thickness lip defects with a size up to 50% of the lip can be closed primarily with better functional and aesthetic outcome and the use of a local flap in such cases can be avoided. This is especially true for elderly patients.

Lip advancement and Estlander flap are other good techniques for small to medium sized full thickness lip defects with good competence, better vermilion match, near anatomical closure, less scarring, acceptable stoma size, and minimal or no donor site morbidity. However, blunting or rounding of commissure that will need future commissuroplasty, hypoesthesia, delayed return of sensation, and asymmetry of the lip are notable drawbacks of Estlander flap. Bektas et al. [12] did transverse lip advancement flap in 10 patients and concluded good functional and aesthetic outcome in all patients. Blunting of commissure, microstomia, and prolonged denervation (6–18 months) are the main shortcomings of Estlander flap [13]. Husein-ElAhmed et al. [14] highlighted that Estlander flap is associated with better camouflage in terms of aesthesis and scar visibility and good sensory recovery in 3 months. Blunting of commissure mostly improved to its normal counterpart level and commissuroplasty is usually not required [13].

Karapandzic flap is a viable option for medium to large sized full thickness defects, especially in those patients with good skin laxity and if other flaps are not an option. It provides single stage reconstruction with good oral competence and normal sensation but microstomia and lip asymmetry are notable. According to Dadhich et al. [15], Ebrahim et al. [16], and Coppit et al. [17] it is a single stage flap which is mostly used for the lower lip but can be used for the upper lip. It has good sensation and oral competence but is associated with microstomia which is problematic in patients with denture and asymmetry of the lip and blunting of commissure. This flap also needs meticulous surgical technique for dissection of neurovascular bundle and orbicularis oris near commissure.

Nasolabial flap can be used for large full thickness lip defects in patients where microstomia cannot be tolerated and for commissural defects with buccal mucosal lesion which need cover after excision. However, oral incompetence occurs in commissural defects which may improve over time. Vermilion mismatch is appreciable and the flap may need thinning to match the remaining lip tissue bulk. Central defect and single lip lateral defect usually don’t have problem of incompetence. With this flap, there is prolonged hypoesthesia. Donor site scar is better camouflaged but standing cutaneous deformity can occur at donor site that may improve over time or may need correction. This flap can be used with other variation like Fujimori Gate flap [18], islanded nasolabial flap, bilateral nasolabial flap with myomucosal advancement [19], for complete lip loss.

Free flaps like free radial artery forearm flap with or with or without palmaris longus sling are reserved for large full thickness defects like total lip defect, lip defect with buccal mucosal defect and where local tissue is not available for reconstruction and where microstomia cannot be tolerated. Lip defect where commissure is involved is associated with oral incompetence. However, central and whole lip defect where PL sling was incorporated had good oral competence. The flap is bulky and vermilion mismatch is there along with asymmetry in labial mobility. However, microstomia is not a problem with adequately sized flap. There is always prolonged hypoesthesia and duration of return of sensation depends upon whether sensory nerve coaptation is done or not. One of our patients had static two-point discrimination at 10 mm after 2 years in whom sensory nerve coaptation was not done. Bulky flaps need staged thinning and correction to match vermilion thickness. Commissural defects need future commissuroplasty. donor site needs skin grafting if primary closure cannot be done. Ebrahimi et al. [20] reviewed the literature and reported that free flap is a good option for full thickness whole lip defect reconstruction when local tissue is unavailable but its functional and aesthetic outcomes are limited due to difference in donor tissue color, elasticity and texture and bulk. They also reported that reconstruction with FRAFF has limitation like numbness, drooling of saliva and competence. They also reported that FRAFF with palmaris longus sling reconstruction is a dynamic and non-sensory reconstruction. Özdemir et al. [21] performed sensate FRAFF with PL sling free mucosal graft for vermilion reconstruction in 17 patients and demonstrated that all patients had good oral competence and started to regain sensation 4 to 6 months after surgery and had acceptable aesthetic results. 

## Conclusion

Functional and aesthetic outcome are good with local lip flap reconstruction. These are even better for smaller defects and central lip defects. Sensory recovery is good and almost complete with local lip flaps over a period of time and is better compared to regional and distant flaps. Commissural defects treated with lip flaps have good oral competence. Microstomia occurs after local lip flap reconstruction. However most patients are not bothered if it is not severe and it can be corrected by commissuroplasty. Posttraumatic defects and defects where post-procedure radiotherapy is not required can be reconstructed with local flaps. However, in cases where postoperative radiotherapy may be required, it is better to avoid microstomia by adding tissue in the form of regional or distant (free) flap during reconstruction, as microstomia may be worsened post radiotherapy. Finally, defect size, defect location, availability of local tissue, patients’ comorbid condition, need for postoperative radiotherapy, and patient’s informed consent and preference decide on the choice of the reconstruction procedure.

## Notes

### Competing interests

The authors declare that they have no competing interests.

### Ethics

The study was approved by the institutional ethics committee. Informed consent for inclusion in the study and use of patients' photographs was obtained.

## Figures and Tables

**Table 1:**
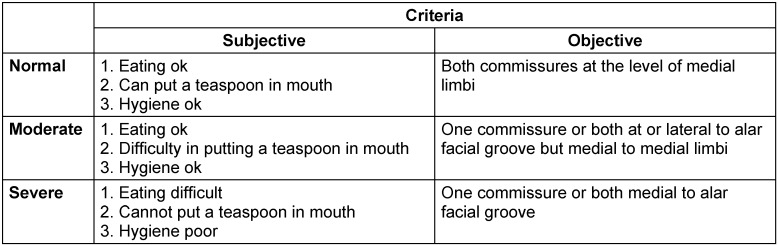
Criteria for microstomia

**Table 2:**
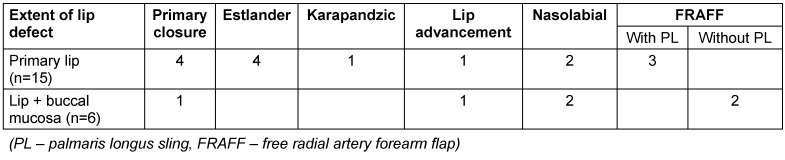
Procedure according to extent of lip defect

**Table 3:**
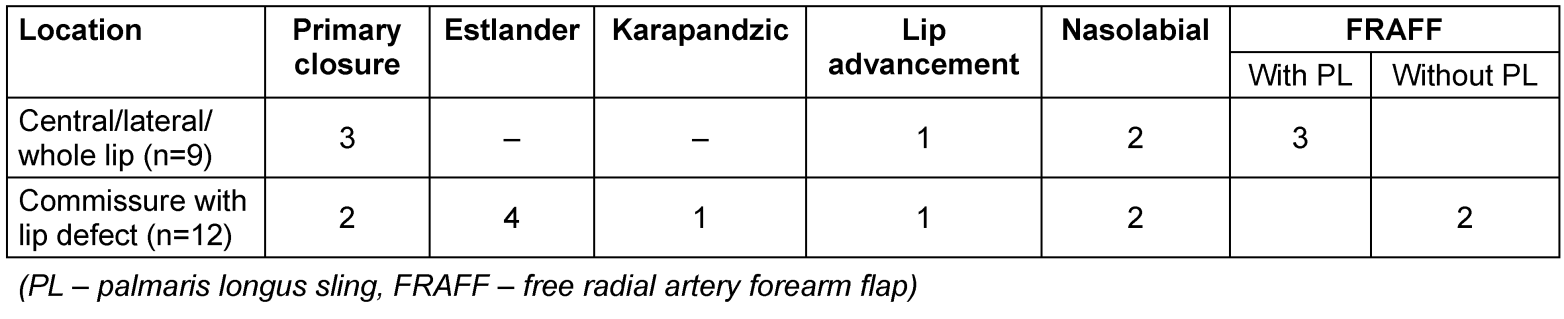
Procedure according to defect location

**Table 4:**
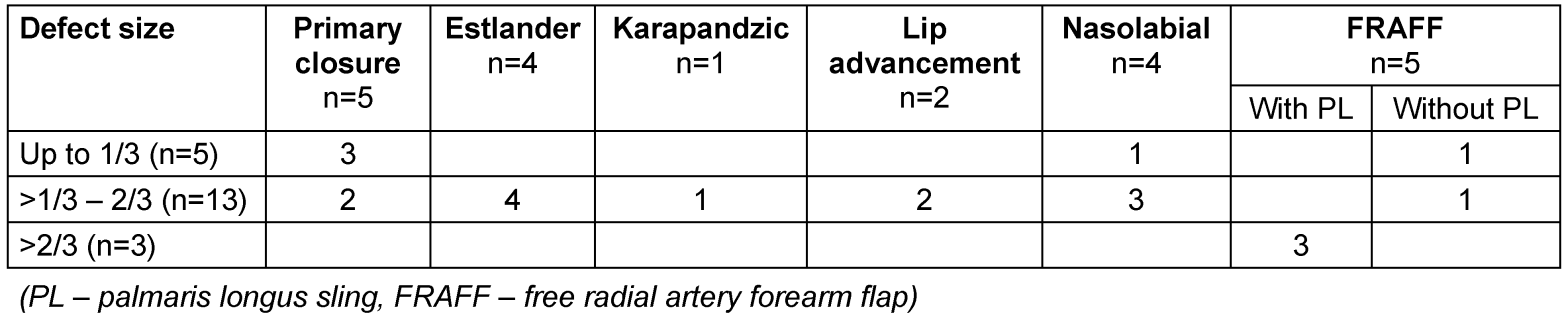
Patient and procedure distribution according to defect size

**Table 5:**
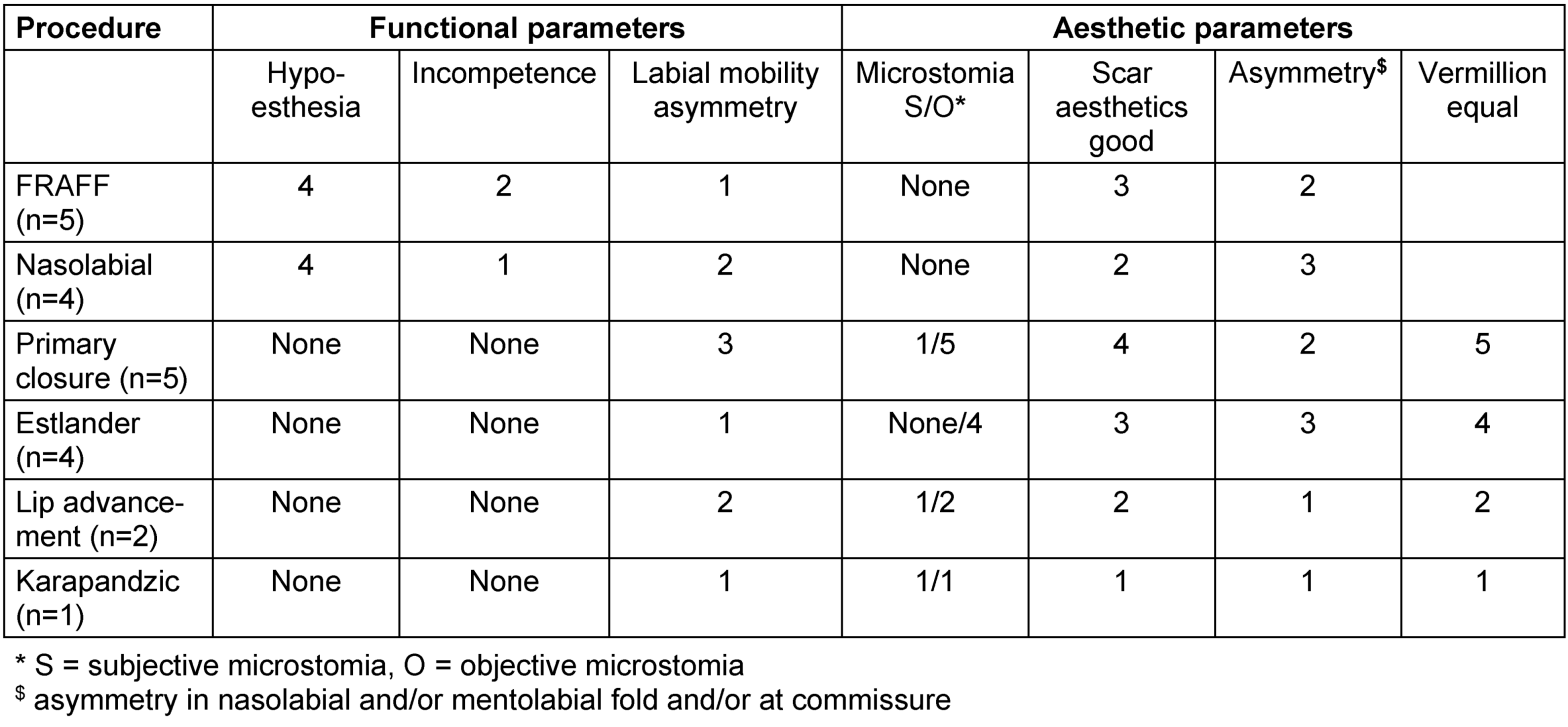
Functional and aesthetic outcome of the different procedures
